# Psychosocial and Social Security Risks Linked to Vaccine Misinformation in Romania: Implications for Vaccination Acceptance and Public Policy

**DOI:** 10.3390/bs16040595

**Published:** 2026-04-16

**Authors:** Flavius Cristian Mărcău, Cătălin Peptan, Olivia-Roxana Alecsoiu, Marian Emanuel Cojoaca, Alina Magdalena Musetescu, Genu Alexandru Căruntu, Alina Georgiana Holt, Lorena Duduială Popescu, Costina Sfinteș, Victor Gheorman

**Affiliations:** 1 Faculty of Educational Sciences, Law and Public Administration, “Constantin Brâncuși” University of Târgu Jiu, 210185 Târgu Jiu, Romania; flavius.marcau@e-ucb.ro (F.C.M.); catalin.peptan@e-ucb.ro (C.P.); olivia.alecsoiu@e-ucb.ro (O.-R.A.); alina.holt@e-ucb.ro (A.G.H.); 2 National Health Insurance House (CNAS), Titu Maiorescu University, 040051 Bucharest, Romania; marian.cojoaca@utm.ro; 3 Victor Babeș Hospital for Infectious and Tropical Diseases, 030303 Bucharest, Romania; 4 Faculty of Medicine, Titu Maiorescu University, 040051 Bucharest, Romania; 5 Faculty of Economics, “Constantin Brâncuși” University of Târgu Jiu, 210185 Târgu Jiu, Romania; genu.caruntu@e-ucb.ro (G.A.C.); lorena.duduiala-popescu@e-ucb.ro (L.D.P.); 6 Institute of Research, Development, and Innovation, “Constantin Brâncuși” University of Târgu Jiu, 210185 Târgu Jiu, Romania; costina.sfintes@e-ucb.ro; 7 Department of Psychiatry, University of Medicine and Pharmacy of Craiova, 200349 Craiova, Romania; victor.gheorman@umfcv.ro; 8 Department of Psychiatry I, Craiova Clinical Neuropsychiatry Hospital, 200473 Craiova, Romania

**Keywords:** vaccination decisions, fake news, disinformation, public health, Romania, pandemic risk perception, social security

## Abstract

This study examines the influence of misinformation on vaccination decision-making and the perception of social security in Romania in the context of potential future pandemics. Using a survey-based design, data were collected through an online questionnaire administered to a sample of 1005 respondents. The analysis employed descriptive and inferential statistical methods, including chi-square tests, ANOVA, Kruskal–Wallis tests, principal component analysis (PCA), K-means clustering, random forest models, and Spearman correlations. The results indicate statistically significant associations between belief in misinformation and vaccination attitudes (*p* < 0.001), with moderate effect sizes. Effect size estimates indicated small-to-moderate associations (e.g., Cramér’s V up to 0.371 for key demographic differences, and Kendall’s W = 0.273 for the increase in willingness across the three severity scenarios). Individuals with higher levels of education, urban residence, and younger age were more likely to report higher willingness to vaccinate, whereas respondents from rural areas and those with lower educational levels showed greater susceptibility to misinformation. In addition, risk perception was significantly associated with vaccination intention, which increased as the severity of hypothetical pandemic scenarios intensified. Predictive modeling identified specific misinformation beliefs—particularly those related to vaccine safety and natural immunity—as key factors associated with vaccination decisions. These findings suggest that misinformation is strongly associated with both individual vaccination behavior and broader perceptions of social security.

## 1. Introduction

In recent decades, rapid epidemiological transformations have placed unprecedented pressure on public health systems, and the COVID-19 pandemic has demonstrated how quickly prevention and intervention structures can react and adapt (Fusillo et al., 2019; Lupu et al., 2016). However, this experience has also highlighted significant vulnerabilities, such as the need to reinforce the importance of vaccination in controlling infectious diseases and combatting a continuously expanding phenomenon: online disinformation (Bianchi & Tafuri, 2023). In a digital space where information circulates rapidly, individuals are exposed to numerous contradictory messages, which directly influence decisions related to both individual and collective health (Cmeciu, 2023).

In the context of this study, the concept of social security is understood not strictly in an institutional sense, but as a multidimensional construct encompassing perceived societal stability, trust in public health institutions, and the population’s sense of protection against large-scale health threats. More specifically, it reflects the extent to which individuals believe that authorities are capable of managing health crises effectively and ensuring collective safety, as trust in institutions and perceptions of government response have been shown to significantly influence public health behaviors and vaccination decisions (G. Chen et al., 2024; Savoia et al., 2022). Furthermore, higher levels of institutional trust have been consistently associated with increased perceived safety and reduced vaccine hesitancy (X. Chen et al., 2022; Huang et al., 2025).

At the same time, it is important to differentiate between several related concepts that are often used interchangeably. In this study, misinformation refers to false or inaccurate information shared without necessarily intending to deceive, whereas fake news denotes deliberately fabricated or manipulated content designed to mislead, both of which have been shown to influence public attitudes and health-related behaviors (Allen et al., 2024; Lazer et al., 2018). In contrast, conspiracy beliefs represent structured interpretative frameworks that attribute hidden intentions and coordinated actions to powerful actors, often rejecting official explanations, and are strongly associated with vaccine refusal and distrust in authorities (Douglas & Sutton, 2023).

These distinctions allow for a clearer theoretical positioning of the study within established frameworks of vaccine hesitancy and risk perception, where exposure to misinformation, endorsement of conspiracy beliefs, and erosion of institutional trust are considered key determinants of vaccination behavior. Empirical evidence shows that misinformation endorsement, lower institutional trust, and lower perceived risk are consistently associated with higher vaccine hesitancy (Carosella et al., 2024). This approach is further supported by theoretical perspectives from health behavior models and risk perception theory, which emphasize the role of perceived threats, trust in institutions, and information exposure in shaping individual decision-making (Majid et al., 2022). Additionally, research highlights that vaccine hesitancy is a multifactorial phenomenon emerging from the interaction between cognitive, social, and institutional factors (Kadir & Ying, 2024; Zhao et al., 2024).

In addition, misinformation theory highlights how repeated exposure to false or misleading information is associated with changes in attitudes and behaviors, particularly in uncertain and high-risk contexts such as pandemics (Kimotho, 2025; Nah et al., 2023). Together, these frameworks provide a conceptual foundation for understanding how misinformation and sociodemographic factors interact in shaping vaccination-related attitudes.

Despite the availability of advanced communication technologies and nearly unlimited access to data, vaccine hesitancy remains a persistent phenomenon (Valeanu et al., 2023). International studies indicate that continuous exposure to false or distorted information erodes trust in medical systems and public health policies, and Romania is no exception. In certain regions and sociodemographic groups, obstacles to accessing credible sources facilitate the spread of alarmist narratives, amplifying skeptical or even radical attitudes toward vaccination (Jain et al., 2022; Nicholls et al., 2024). Furthermore, weak communication between responsible institutions and historical distrust in authorities create fertile ground for conspiracy theories, increasing the population’s vulnerability in the event of future epidemiological crises (Lazarus et al., 2024; Mărcău et al., 2022b; Peptan et al., 2022).

At the European level, vaccine hesitancy has been recognized as a persistent and multifaceted challenge, extending beyond the specific context of the COVID-19 pandemic (Vuolanto et al., 2024; Barbieri et al., 2024). While the pandemic amplified public debates and polarization around vaccination, several studies indicate that hesitancy reflects a broader pattern linked to trust in institutions, perceived risks, and exposure to misinformation (Carrieri et al., 2023; Jennings et al., 2021a). Compared to the European average, Romania has consistently reported lower vaccination coverage rates, not only during the COVID-19 campaign but also in routine immunization programs (Włodarska et al., 2024; World Health Organization, 2025). This suggests that vaccine hesitancy in Romania is not exclusively a pandemic-related phenomenon but rather part of a more general and enduring trend affecting vaccination behaviors (Dascalu et al., 2025).

In Romania, the COVID-19 vaccination campaign revealed major difficulties: the lack of effective coordination and well-structured communication strategies led to distrust and a low immunization rate. Large stocks of unused vaccines result in financial and logistical losses (HotNews, 2025a), reinforcing the perception of poor crisis management and undermining institutional credibility (Dascalu et al., 2021; HotNews, 2025b). In this fragile climate, the influence of misinformation and conspiracy theories intensified, obstructing efforts to achieve sufficient vaccine coverage to limit the spread of the virus (Jennings et al., 2021a; Mureşan & Salcudean, 2022). Against this backdrop, the risk of diseases previously considered under control, such as poliomyelitis, has increased, as the World Health Organization continues to classify Romania as a high-risk country due to insufficient vaccine coverage (World Health Organization, 2025). The absence of a firm prevention policy and a strategy to persuade citizens, combined with a tendency to underestimate traditional epidemiological threats, may pave the way for new outbreaks with potentially severe consequences for the healthcare system (Dascalu et al., 2025; P. Sandu et al., 2021).

Although international research has extensively explored the relationship between misinformation and vaccine hesitancy, fewer studies have simultaneously examined the interaction between misinformation, sociodemographic factors, and hypothetical pandemic scenarios, particularly in Eastern European contexts (Jennings et al., 2021b; Lazarus et al., 2024; Nicholls et al., 2024; Palmer & Gorman, 2025). Moreover, most existing studies (F. Zhang et al., 2022; Allen et al., 2024; Barqawi et al., 2024; Rahbeni et al., 2024) focus on real-time crisis situations, such as COVID-19, with limited attention to how these dynamics may evolve in future pandemic contexts. By integrating these dimensions, the present study addresses this gap and provides a more comprehensive understanding of vaccination decision-making under varying levels of perceived risk.

Vaccination remains one of the most effective tools for preventing and controlling infectious diseases (Shattock et al., 2024; Montero et al., 2024), yet the COVID-19 pandemic highlighted that the success of vaccination programs depends not only on biomedical innovation and vaccine availability, but also on public perception, trust, and willingness to accept immunization (Garett & Young, 2021; Hicar, 2022; MacDonald, 2020; Rahbeni et al., 2024). Across diverse contexts, vaccine hesitancy has been consistently described as a multidimensional phenomenon shaped by interacting social, psychological, political, and economic factors, with the post-pandemic period further amplifying polarization and distrust in health communication (Badur et al., 2020; Cagnotta et al., 2025; Neely et al., 2021; Ortiz-Prado et al., 2025; Tejan, 2021). Evidence synthesized in recent umbrella and systematic reviews indicates that exposure to misinformation is systematically associated with lower vaccine confidence and higher hesitancy, and that these effects are not limited to the COVID-19 vaccine but can spill over into attitudes toward routine immunization and other vaccination programs (Allen et al., 2024; Rahbeni et al., 2024; UNICEF, 2023; F. Zhang et al., 2022).

This relationship between the informational environment and vaccination behavior is particularly salient in digital ecosystems where contradictory, emotionally charged, or conspiratorial messages circulate quickly and can undermine institutional credibility (Allen et al., 2024; Barqawi et al., 2024; F. Zhang et al., 2022). Recent research also emphasizes that misinformation operates through identifiable mechanisms—such as fear-based safety narratives, distrust in institutions and industry, and the idealization of “natural” alternatives—suggesting the need for tailored counter-messaging rather than generic information campaigns (Barqawi et al., 2024; Cagnotta et al., 2025). In Romania, vaccine hesitancy and institutional distrust have been repeatedly discussed as structural challenges, and post-pandemic analyses highlight how misinformation and weak crisis communication can intensify skepticism toward public health measures (Dascalu et al., 2025; Mărcău et al., 2022a; P. Sandu et al., 2021; World Health Organization, 2025). Because some of the Romanian patterns may be stronger than those typically reported in Western European samples, understanding how misinformation interacts with sociodemographic inequalities and trust in institutions remains crucial for policy design (Dascalu et al., 2025; Dumitra et al., 2024; Mărcău et al., 2022a).

Sociodemographic determinants are consistently linked to vaccination attitudes. Age, education, income, and place of residence are frequently reported as predictors of vaccine acceptance, often through differences in access to reliable information, health services, and levels of institutional trust (Bocquier et al., 2018; Jin et al., 2022; Machida & Inoue, 2023; Manolescu et al., 2022; Mendoza, 2020). These patterns are also discussed in relation to differential exposure to misinformation and varying capacities for evaluating health claims, which may translate into unequal vulnerability to conspiratorial content (Jin et al., 2022; Machida & Inoue, 2023; Motiejūnaitė et al., 2025). At the same time, risk perception remains a central driver of preventive behavior: when perceived threat increases, willingness to adopt protective actions, including vaccination, tends to rise, though misinformation may still attenuate acceptance even under high-risk conditions (Hilverda & Vollmann, 2021; Martinelli & Veltri, 2023; Sorrentino et al., 2025).

From a public health perspective, the literature converges on the importance of communication strategies that are clear, timely, and evidence-based, especially in environments where misinformation spreads rapidly (Jennings et al., 2021a; Lamot & Kirbiš, 2024; Pluviano et al., 2017; Ruggeri et al., 2024). Interventions that strengthen digital and health literacy are increasingly considered essential to improving the public’s ability to evaluate online health information and reduce susceptibility to false claims (Biasio, 2017; Marzo et al., 2022; Meier et al., 2026; Motiejūnaitė et al., 2025). In addition, collaboration between health institutions, schools, community leaders, and credible public figures is frequently highlighted as a practical route to rebuilding trust and supporting vaccine acceptance, particularly in communities where institutional trust is fragile (Biasio et al., 2024; Casigliani et al., 2020; Dib et al., 2021; Wilson et al., 2017). Taken together, these strands of evidence indicate that vaccine hesitancy emerges from a complex interplay between misinformation exposure, sociodemographic inequalities, and risk perception, and that effective responses require integrated approaches combining targeted communication, education, and trust-building measures (Badua et al., 2022; Barqawi et al., 2024; Ruggeri et al., 2024; F. Zhang et al., 2022).

In this context, the present study aims to investigate in depth the dynamics of vaccination decisions in Romania, considering both the specific impact of disinformation and the persistent hesitancy of a significant portion of the population. Through a detailed analysis of sociodemographic factors and the informational environment, this research seeks to provide practical recommendations for public policies that could improve the management of future crises and restore trust in protective measures against infectious diseases.

The integrated approach presented in this study treats disinformation both as a social phenomenon with historical and cultural roots and as a specific challenge of the digital era. By analyzing both vaccination behaviors and the beliefs that influence them, this endeavor aims to lay the foundation for future prevention strategies, not only to address the threats of a potential new pandemic but also to combat the harmful effects of fake news in society.

### Research Objectives and Hypotheses

Objectives

1. Identifying the sociodemographic variables that influence vaccination decisions in a pandemic context (O1).
2. Analyzing the impact of false information on attitudes toward vaccination (O2).
3. The development of proposals for public policies and communication campaigns aimed at increasing vaccine acceptance and reducing the negative effects of disinformation (O3).

Hypotheses

The main hypothesis of the study is that, in a possible future pandemic context, vaccine hesitancy will be amplified by a high level of trust in “fake news” disseminated online. Specifically, we hypothesize that an increased level of disinformation is associated with a decrease in vaccine acceptance (H1). Furthermore, we expect that certain sociodemographic factors significantly influence susceptibility to disinformation and, consequently, the decision to vaccinate (H2).

## 2. Materials and Methods

### 2.1. Sample Description

The study was conducted on a sample of 1005 respondents from Romania drawn from various sociodemographic categories. This sample includes individuals of different ages, with distributions based on gender, place of residence (urban or rural), education level, employment sector, and income. The diversity of the sample, represented in Table 1, allows for a detailed analysis of attitudes toward vaccination and the factors influencing the decision to vaccinate in a pandemic context.

The sample was obtained using a non-probabilistic, convenience sampling strategy based on voluntary participation. Eligibility criteria included age ≥ 18 years, permanent residence in Romania, and consent to participate. Because recruitment was conducted online, the sample should be interpreted as heterogeneous but not statistically representative of the Romanian population. Therefore, generalizations to the national level should be made cautiously, with attention to potential selection bias.

The target sample size (N = 1005) was selected to support (i) adequate precision for estimating population proportions and (ii) stable subgroup comparisons across key sociodemographic strata. In survey research, a sample size of around ~1000 is commonly used as a practical benchmark for large populations when aiming for a small margin of error under conservative assumptions (Krejcie & Morgan, 1970). In addition, contemporary methodological guidance emphasizes that sample size planning should consider precision, feasibility, and the intended analytic strategy, especially for cross-sectional surveys that compare groups and estimate associations (Ranganathan et al., 2024).

### 2.2. Research Instrument

To collect the data, a structured questionnaire was created via Google Forms (Google LLC, Mountain View, CA, USA). This instrument included demographic questions (age, gender, place of residence, education, employment sector, income) as well as specific questions regarding attitudes toward vaccination and perceptions of disinformation. The questionnaire comprises both multiple-choice questions (e.g., Q1—personal values; Q2–Q7—attitudes toward mandatory and optional vaccines; and Q8–Q10—willingness to vaccinate on the basis of pandemic severity) and Likert scale questions (FN1–FN15) to assess agreement or disagreement with various statements, including those reflecting conspiracy theories and fake news.

A structured questionnaire was chosen because it enables standardized measurement across respondents, supports comparability across sociodemographic categories, and facilitates reproducible hypothesis testing in survey-based health research (Dillman et al., 2014). Because the questionnaire was administered online, reporting also follows established guidance for web-based surveys that emphasizes transparency in recruitment, consent, survey administration, and response handling (Eysenbach, 2004).

The FN1–FN15 items were constructed based on recurrent misinformation themes identified in the literature on vaccine hesitancy and public health communication, particularly those related to vaccine safety, conspiracy narratives, and distrust in pharmaceutical companies. Although these items were not directly adapted from a single previously validated scale, they reflect widely documented categories of health-related misinformation (Kisa & Kisa, 2024; Jennings et al., 2021b; Palmer & Gorman, 2025).

To assess the internal consistency of the scale, a reliability analysis was conducted. The results indicated an excellent level of internal consistency (Cronbach’s alpha = 0.977), suggesting that the items capture a highly coherent underlying construct related to susceptibility to misinformation and conspiracy beliefs. This high reliability value also supports the use of a composite misinformation index in subsequent analyses.

However, while the high Cronbach’s alpha supports internal consistency (Nunnally & Bernstein, 1994; Bujang et al., 2024), it should not be interpreted as evidence of construct validity. The very high value (α = 0.977) may partly reflect item redundancy—that is, several items may capture overlapping aspects of the same belief cluster—rather than comprehensive coverage of the misinformation construct. Because the FN1–FN15 scale was not adapted from a previously validated instrument and has not undergone formal construct validation (e.g., confirmatory factor analysis against an external criterion such as the Vaccine Conspiracy Beliefs Scale; Shapiro et al., 2016), the composite scores should be interpreted as reflecting a coherent but not formally validated dimension of misinformation susceptibility.

Before conducting principal component analysis, the adequacy of the data was assessed. The Kaiser–Meyer–Olkin measure indicated excellent sampling adequacy (KMO = 0.971), and Bartlett’s test of sphericity was statistically significant (χ^2^(105) = 18,571.92, *p* < 0.001), supporting the suitability of the data for dimensionality reduction.

### 2.3. Data Collection Procedure

The questionnaire [Appendix A] was administered online using Google Forms. Participation was voluntary and anonymous, and no incentives were offered. Informed consent was provided in the survey preamble, and respondents could proceed only after confirming that they were at least 18 years old, had permanent residence in Romania, and agreed to participate. No identifying information was collected, and responses were used exclusively for research purposes.

Recruitment was conducted through online distribution channels, including social media platforms and email networks, enabling access to respondents from diverse sociodemographic backgrounds (Sledzieski et al., 2023; Torrejón-Guirado et al., 2024). The sampling strategy can be characterized as non-probabilistic and based on convenience and voluntary participation (Ahmed, 2024; Pew Research Center, 2023). As a result, the study may be affected by selection bias, as individuals with better internet access and higher digital literacy are more likely to participate, while groups with limited access to technology—particularly from disadvantaged or rural settings—may be underrepresented. Therefore, the results should be interpreted with caution in terms of representativeness and generalizability to the broader Romanian population.

### 2.4. Data Analysis Methods

Data analysis was conducted via a combination of statistical methods designed to capture both general trends and significant relationships between variables, consistent with methodological recommendations for observational and cross-sectional research that combine descriptive summaries with inferential and multivariable approaches to estimate associations and assess robustness (Maier et al., 2023; Pérez-Guerrero et al., 2024). The data were processed in IBM SPSS 26 (IBM Corp, Armonk, NY, SUA) (Kaid Al-Mohani et al., 2026; Shahrul & Syed Mohamed, 2024) and Microsoft Excel (Microsoft Corporation, Redmond, WA, USA), with initial exploration including frequency and distribution analysis. Relationships between variables were examined via chi-square tests, ANOVA, and Kruskal–Wallis tests, depending on the characteristics of the data. To identify distinct respondent typologies, principal component analysis (PCA) and K-means clustering were applied, whereas random forest models were used to highlight the most influential factors in the vaccination decision. Additionally, Spearman correlations were used to assess the extent to which exposure to disinformation affects attitudes toward vaccination.

Although multiple analytical techniques were employed, each was selected to address a specific research question aligned with the study objectives. Descriptive and inferential tests (chi-square, ANOVA, Kruskal–Wallis) served O1 by testing whether vaccination attitudes and misinformation susceptibility differ significantly across sociodemographic groups, as these methods are widely recommended for examining associations between categorical variables and identifying statistically significant differences across independent groups (Chicco et al., 2025; S. W. Lee, 2022; Mabizela, 2025). Principal component analysis and K-means clustering served O2 by identifying latent attitudinal dimensions and segmenting respondents into empirically derived typologies based on their susceptibility to misinformation and vaccination attitudes (Gao et al., 2023; Jolliffe & Cadima, 2016; Şan & Göçken, 2025; Veri, 2025). Random forest models complemented these by quantifying the relative predictive importance of individual misinformation items in a non-linear framework (O’Connell et al., 2025; Rothacher & Strobl, 2024), while Spearman correlations assessed the strength and direction of associations between misinformation endorsement and vaccine acceptance (Tu et al., 2025; Wisniewski & Brannan, 2024).

Finally, multinomial logistic regression integrated sociodemographic and attitudinal predictors into a single multivariable model, providing adjusted estimates relevant to both O1 and O2. Together, these methods form a layered analytical strategy in which exploratory, confirmatory, and predictive approaches triangulate the findings and increase the robustness of the conclusions. The core analytical components are the inferential group comparisons (chi-square, ANOVA, Kruskal–Wallis) and the correlation analyses (Spearman), which directly test the study hypotheses. PCA with K-means clustering and random forest models serve a complementary role by providing exploratory pattern identification and variable importance ranking, respectively, while multinomial logistic regression offers an integrative multivariable perspective. This multi-method design also directly informs O3 by identifying which population segments and which specific misinformation themes should be prioritized in public health communication strategies.

## 3. Results

### 3.1. Trust in Conspiracy Theories

In response to the question “What values guide your behavior?”, scientifically proven achievements over time were selected by 84.8% of the participants, whereas religious ideas and concepts were chosen by 15.15%. In addition to statistical significance, effect sizes were reported where appropriate to assess the practical relevance of the findings. Participants’ trust in “fake news” information (FN1–FN15) is presented in Table 2.

Across all fake news items, between 12.0% and 16.0% of respondents selected a neutral rating (Rating 3). Ratings 4–5 ranged from 6.0% to 22.9% across the FN items (Table 2).

### 3.2. Attitudes Toward Childhood and Optional Vaccines

Table 3 presents vaccination attitudes according to key sociodemographic variables. Given the density of the full cross-tabulation, a simplified version highlighting the most statistically relevant comparisons is presented below. The complete breakdown by all sociodemographic categories is available in Supplementary Table S1.

Cramér’s V values are reported in Table 3 for each association. For COVID-19 vaccination (Q6), V values were 0.294 (Gender), 0.366 (Age), 0.242 (Environment), and 0.371 (Income). Among respondents who received the COVID-19 vaccine, 84.2% opted for an mRNA-based vaccine.

The influence of fake news on the acceptance of mandatory and optional vaccines was examined through a multi-method analytical approach. The clustering and group-difference results are presented in Table 4a, while the predictive modeling and correlation results are presented in Table 4b.

Positive Spearman ρ values indicate that higher fake news belief scores are associated with lower vaccine trust (Q2 and Q4 coded 1 = Yes, 2 = No).

Table 4a reports the clustering and group-difference analyses, and Table 4b reports the top Random Forest and Spearman results. All reported associations were statistically significant (*p* < 0.001).

### 3.3. Willingness to Vaccinate in the Event of a New Pandemic

a. Scenarios by severity

Table 5 presents vaccination acceptance across three hypothetical pandemic severity scenarios, stratified by sociodemographic variables.

A Friedman test for repeated measures revealed a statistically significant difference in vaccination willingness across the three pandemic scenarios (χ^2^(2) = 548.15, *p* < 0.001, Kendall’s W = 0.273), indicating a small-to-moderate effect. Mean willingness scores increased progressively from Q8 (milder scenario) through Q9 (COVID-19-like) to Q10 (more severe scenario).

ANOVA tests confirmed that all demographic variables significantly influenced vaccination intentions across all three scenarios. Gender consistently showed the largest effect sizes (Q8: F = 96.21, η^2^ = 0.088; Q9: F = 142.43, η^2^ = 0.124; Q10: F = 151.91, η^2^ = 0.132). Income effects strengthened with pandemic severity (Q8: η^2^ = 0.081; Q9: η^2^ = 0.129; Q10: η^2^ = 0.141). The lowest acceptance rates were consistently found among respondents aged 18–25, females, those in rural areas, and those in the lowest income bracket.

The influence of fake news on vaccination intentions across the three pandemic scenarios was examined using multiple analytical approaches. A simplified version focusing on the most influential variables is presented below (Table 6). The complete results are reported in Supplementary Table S3.

K-Means clustering identified two groups across all three pandemic scenarios: a skeptical group with high fake news belief scores and a pro-vaccination group with low scores. The Kruskal–Wallis test confirmed significant between-group differences in vaccination intent, with effect sizes increasing with pandemic severity (ε^2^ = 0.288 for Q8, ε^2^ = 0.343 for Q9, ε^2^ = 0.383 for Q10). The Random Forest model identified FN14 (“Natural immunity is always safer and more effective than any vaccine”) as the most important predictor for Q8 (15.3%) and Q10 (13.6%). Spearman correlations confirmed that all FN1–FN15 variables were significantly negatively correlated with vaccination intent across all three scenarios (all *p* < 0.001), with the strongest correlations recorded for FN12, FN14, FN13, and FN11.

b. Preferences for the type of vaccine

Table 7 presents the results of multinomial logistic regression and Random Forest analyses examining the association between demographic factors, fake news beliefs, and vaccine type preferences (Q11). The reference category was mRNA vaccines.

Gender was the strongest predictor of vaccine refusal (β = 1.433, 95% CI [1.087, 1.780]), indicating that women were significantly more likely than men to refuse vaccination relative to choosing an mRNA vaccine. Income was a significant negative predictor for both vaccine refusal (β = −0.389, 95% CI [−0.543, −0.235]) and preference for inactivated/subunit vaccines (β = −0.318, 95% CI [−0.582, −0.053]), suggesting that higher income was associated with greater preference for mRNA vaccines. mRNA vaccines were preferred by 50.4% of respondents, while 26.7% expressed unwillingness to vaccinate.

The distribution of preferences regarding vaccine type (Q11) should be interpreted with caution. Some response categories recorded very low frequencies (e.g., inactivated/subunit vaccines: 6.1%), which limits the robustness of comparisons between groups. Therefore, the interpretation focuses primarily on broader attitudinal patterns rather than detailed categorical distinctions across all response options.

## 4. Discussion

In relation to the study objectives, the results provide convergent evidence across analytical layers. For O1, vaccination attitudes and intentions differed systematically by sociodemographic factors, with gender and income showing the largest effects across key outcomes (e.g., moderate Cramér’s V values in chi-square analyses and substantial coefficients with 95% CIs in the multinomial model). For O2, misinformation belief scores were consistently associated with lower vaccine acceptance across mandatory, optional, and scenario-based intentions, and predictive modeling highlighted a small set of misinformation themes (vaccine safety and natural immunity narratives) as the most influential contributors to hesitancy. For O3, these patterns support the development of targeted, segment-specific public health communication and digital health literacy interventions, aligned with recent evidence on countering health misinformation and improving trust-oriented messaging (Malik et al., 2023; Ruggeri et al., 2024), as reported in Table 3, Table 4, Table 5, Table 6 and Table 7 and Supplementary Tables S1–S3.

However, several important caveats apply. Given the cross-sectional design, the identified relationships should be interpreted as associations rather than causal effects. Although many associations reached statistical significance, most effect sizes were moderate (η^2^ ranging from 0.041 to 0.144; Cramér’s V from 0.091 to 0.371), indicating that misinformation is an important but not exclusive determinant of vaccination attitudes. To illustrate the practical relevance of these magnitudes: the Cramér’s V of 0.371 for income and COVID-19 vaccination corresponds to approximately 14% of shared variance, meaning that income category alone accounts for a substantial proportion of the variation in vaccination status—a noteworthy magnitude in a context where vaccination behavior is shaped by dozens of competing factors. Similarly, the η^2^ values for gender effects across pandemic scenarios (0.088–0.132) indicate that gender differences explain roughly 9–13% of the variance in vaccination willingness, a magnitude that is directly actionable for designing gender-targeted health communication. The absolute gap between the lowest and highest income groups in COVID-19 vaccine uptake (46.6% vs. 87.9%—a 41.3 percentage-point difference) further underscores that these statistically moderate effects translate into large real-world disparities in vaccination coverage (Mesquida & Lakens, 2024; Perugini et al., 2025). Future research employing longitudinal or experimental designs would be necessary to establish causal pathways.

### 4.1. Influences of the Sociodemographic Variables

Our analysis revealed that sociodemographic characteristics are essential factors in shaping opinions and behaviors related to vaccination. Essentially, these factors determine both how individuals access and interpret public information and the extent to which they are associated with misleading or alarmist content (Gundogdu, 2020; Islam et al., 2024; Kyprianidou et al., 2023; Włodarska et al., 2020).

Age was significantly associated with both vaccine trust and vaccine type preference. Younger individuals (under 35) showed greater openness to mRNA-based vaccines, possibly reflecting greater familiarity with digital environments and stronger interest in recent scientific developments (Davisson & Hoyle, 2023; Primieri et al., 2023; Tarasi et al., 2023). Older groups preferred vaccines based on traditional methods (viral vectors or inactivated vaccines), suggesting established trust in conventional approaches and, at times, increased caution toward medical innovations (Airapetian et al., 2024; Berk et al., 2024).

Gender was the strongest demographic predictor across nearly all outcomes. Men were consistently more receptive to vaccines than women (e.g., COVID-19 vaccine uptake: 86.7% vs. 60.9%; Cramér’s V = 0.294). These patterns are summarized in Table 3 (see also Supplementary Table S1 for the full cross-tabulation). In the multinomial logistic regression (Table 7), gender was the strongest predictor of vaccine refusal (β = 1.433, 95% CI [1.087, 1.780]). This difference may be attributed to gendered risk perception and differential exposure to contradictory health information. Women may encounter a greater volume of alarmist content, particularly regarding fertility-related misinformation (FN9), which may amplify their reluctance (Ansari et al., 2020; Hallberg et al., 2021; Temuçin & Kızıler, 2024; Vasileva et al., 2022).

The urban–rural divide also played a significant role. Urban residents generally have better access to verified information sources and specialized medical services, fostering more pro-vaccination attitudes (Miao et al., 2024; Saçıkara et al., 2023; Soorapanth et al., 2023). Rural residents, facing more limited resources and weaker informational infrastructure, were more vulnerable to misinformation and consequently more hesitant about vaccines (Cataldi et al., 2021; Liepins et al., 2024). The effect was particularly pronounced for COVID-19 vaccine uptake (54.0% rural vs. 80.2% urban; V = 0.242).

Education and income followed consistent patterns. Higher education was associated with greater ability to distinguish credible information from unsubstantiated claims. Higher income was linked to better access to healthcare services and quality information channels. This gradient was especially strong for COVID-19 vaccination, where acceptance ranged from 46.6% in the lowest income bracket to 87.9% in the highest (V = 0.371). Those with lower incomes were more exposed to misleading messages and generally had limited access to reliable information sources (Fang et al., 2024; Roy et al., 2022; Smith et al., 2023).

These sociodemographic disparities also carry implications for social security as conceptualized in this study. When large population segments—particularly rural, lower-income, and less-educated groups—exhibit lower vaccine acceptance and higher misinformation susceptibility, the collective capacity to respond to large-scale health threats is weakened, undermining the perceived stability and protective function of public health systems (Lazarus et al., 2024; Savoia et al., 2022).

### 4.2. The Role of Risk Perception in Vaccine Acceptance

Beyond sociodemographic factors and misinformation exposure, our study reveals that perceived risk plays a distinct and important role in vaccination decisions. The three hypothetical pandemic scenarios (Q8: mild; Q9: moderate; Q10: severe) allowed us to examine how varying levels of perceived threat influence vaccination willingness independently of misinformation effects.

The results clearly show that perceived pandemic severity increases vaccination intent. The Friedman test (Table 5) confirmed a significant progressive increase in willingness across scenarios (χ^2^(2) = 548.15, *p* < 0.001, Kendall’s W = 0.273). For instance, among respondents aged 18–25, acceptance rose from 16.9% (Q8) to 31.6% (Q10), and among those aged 46–55, from 55.3% to 74.1%. This finding aligns with international research indicating that risk perception is a strong driver of preventive behavior (Hilverda & Vollmann, 2021; Martinelli & Veltri, 2023).

Importantly, this severity-dependent increase in vaccination willingness was observed even among respondents with moderate levels of fake news belief, suggesting that heightened risk perception can partially counteract the negative influence of misinformation on vaccination decisions (Brisbois et al., 2024; Fasiku et al., 2024). However, the Kruskal–Wallis effect sizes increased with pandemic severity (ε^2^ = 0.288 for Q8, 0.343 for Q9, 0.383 for Q10), indicating that the gap between skeptical and pro-vaccination groups also widened as the scenario became more threatening (Table 6, Supplementary Table S3). This means that while higher perceived risk increases overall vaccination willingness, it does not eliminate the influence of misinformation—rather, both factors operate simultaneously and their effects are additive.

Demographic factors also moderated the relationship between risk perception and vaccination intent. Gender effects strengthened with pandemic severity (η^2^ = 0.088 for Q8, 0.124 for Q9, 0.132 for Q10), as did income effects (η^2^ = 0.081 for Q8, 0.129 for Q9, 0.141 for Q10). This suggests that as perceived threat increases, pre-existing sociodemographic inequalities in vaccine access and information quality become more—not less—salient (Doornekamp et al., 2020; Tsang et al., 2022; Wong & Yang, 2023).

From a social security perspective, these findings suggest that perceived pandemic severity alone is insufficient to guarantee collective protection. If misinformation continues to suppress vaccine acceptance among specific demographic groups even under high-threat conditions, the overall resilience of the population to health crises remains compromised (Hilverda & Vollmann, 2021; Sorrentino et al., 2025).

### 4.3. The Impact of Misinformation and Implications for Public Health

Our analyses demonstrate that misinformation has a consistent, substantial, and independent influence on vaccination attitudes across all examined outcomes. Clustering analysis (Table 4a) revealed two distinct groups—a skeptical group with high fake news belief scores and a pro-vaccination group with low scores—and this structure remained stable regardless of pandemic severity (Q8, Q9, Q10). The between-group differences in vaccination intent were large (Kruskal–Wallis H ranging from 289.84 to 385.54, all *p* < 0.001). Cluster contrasts and scenario-level group differences are reported in Table 6 (see Supplementary Table S3 for the complete set of FN items). In this sense, misinformation does not only affect individual vaccination decisions, but also alters the perceived reliability of institutional protection mechanisms, thereby directly undermining the social security dimension.

The most harmful misinformation items, as identified consistently across random forest and Spearman correlation analyses, fall into three thematic categories. The first concerns vaccine safety myths: FN4 (“Vaccines cause autism”; RF importance = 0.0959–0.1023; ρ = 0.508–0.544) and FN13 (“Vaccines contain toxic heavy metals”), which target perceived vaccine harm and safety-related anxieties (Table 4b, Supplementary Table S2). The second involves immune system destruction narratives: FN11 (“Childhood vaccination destroys the immune system”; ρ = 0.517–0.618) and FN12 (“Repeated vaccination leads to complete immune system destruction”) exploit fears about long-term cumulative damage. The third promotes the superiority of natural alternatives: FN14 (“Natural immunity is always safer”; RF importance = 0.153 for Q8, ρ = −0.592–−0.617) and FN10 (“Natural medicine makes vaccines unnecessary”) provide an apparently positive rationale for vaccine refusal. This thematic structure suggests that misinformation does not operate as a monolithic force but rather through distinct psychological mechanisms—fear of harm, distrust of cumulative intervention, and idealization of natural processes—each of which requires a tailored counter-messaging approach (see Table 4a,b and Table 6, with expanded outputs in Supplementary Tables S2 and S3).

From a public health perspective, even moderate effect sizes can translate into meaningful population-level consequences (Mesquida & Lakens, 2024; Perugini et al., 2025). The Spearman correlations between specific misinformation items and vaccine acceptance (ρ = 0.50–0.62) indicate that approximately 25–38% of the variance in vaccine acceptance is shared with endorsement of these specific claims. In practical terms, this means that interventions successfully reducing belief in a small number of targeted misinformation themes—particularly those related to vaccine safety (FN4, FN13), immune system damage (FN11, FN12), and natural immunity superiority (FN14)—could yield appreciable gains in vaccine uptake across large populations. Furthermore, the progressive increase in Kruskal–Wallis effect sizes from Q8 (ε^2^ = 0.288) to Q10 (ε^2^ = 0.383) suggests that the gap between misinformation-endorsing and non-endorsing groups widens as pandemic severity increases, indicating that the public health cost of unaddressed misinformation becomes greater precisely when vaccination is most urgently needed.

While the cross-sectional design precludes causal conclusions, the consistent patterns observed across multiple analytical approaches allow us to outline several evidence-informed policy directions that merit further investigation. These directions are particularly relevant from a social security standpoint, as the erosion of vaccine acceptance driven by misinformation represents not only an individual health risk but also a threat to societal resilience and collective preparedness for future pandemics (Lazarus et al., 2024; Ruggeri et al., 2024).

Firstly, because specific fake news items rather than generalized misinformation were most strongly associated with vaccine hesitancy, counter-messaging campaigns could benefit from developing targeted rebuttal materials for each of the identified claims, using techniques from inoculation theory—that is, pre-emptively exposing individuals to weakened forms of misinformation arguments alongside factual refutations (Camargo et al., 2024; Jones & James, 2021; Larson & Broniatowski, 2021; Neely et al., 2021).

Secondly, the strong gender effect on vaccine refusal (β = 1.433) combined with the role of fertility-related misinformation (FN9) suggests that vaccination campaigns should specifically address concerns frequently raised by women, potentially through partnerships with gynecologists, midwives, and maternal health services (Temuçin & Kızıler, 2024).

Thirdly, the pronounced income gradient in vaccine acceptance (V = 0.371 for COVID-19 vaccination) suggests that free vaccination access points in low-income neighborhoods and the integration of vaccination opportunities into social service delivery points such as employment offices and social aid distribution centers (Fang et al., 2024; Smith et al., 2023) could help reduce barriers to uptake, although this would require confirmation through intervention-based research.

Fourthly, the finding that vaccination willingness increases significantly with perceived pandemic severity (Kendall’s W = 0.273) suggests that public health messaging during future pandemics should clearly communicate evidence-based risk assessments rather than minimizing threats, as risk-downplaying communication may inadvertently reinforce the misinformation narrative that pandemics are exaggerated (Dubé et al., 2020; Hwang, 2020; Okuno et al., 2021; Schell et al., 2023; Welch et al., 2023).

Fifthly, given the significant urban–rural gap (COVID-19 vaccine: 54.0% vs. 80.2%; V = 0.242), community-based digital health literacy workshops could be considered in rural areas, delivered through existing trusted infrastructure (schools, churches, local health centers), with content specifically designed to address the most prevalent fake news items identified in this study. Engaging trusted community figures—family doctors, teachers, religious leaders—as communication intermediaries can help disseminate accurate information in communities where institutional trust is low (Bincoletto et al., 2024; Lewin et al., 2022; Muric et al., 2021; Paladini et al., 2022; Vulpe, 2020).

Finally, digital literacy programs specifically targeting the identification and critical evaluation of health misinformation should be integrated into school curricula and adult education programs, particularly for populations with lower education levels (Mufida & Mulya, 2023; Simon et al., 2023; Thaker & Subramanian, 2021; B. Zhang et al., 2024). Authorities would also benefit from maintaining constant, transparent communication—publishing updated epidemiological data, vaccine efficacy information, and immunization progress reports—to ensure that official sources remain credible alternatives to misinformation (Alsharawy et al., 2021; Gorman, 2021; Ichino, 2022; Ledderer et al., 2026).

While these directions are grounded in the observed associations, they should be interpreted as evidence-informed hypotheses for intervention rather than direct causal prescriptions.

### 4.4. Comparison with Other Studies and Possible Cultural Explanations

The results of this study largely align with international research on vaccine hesitancy, yet they also reveal features attributable to Romania’s specific cultural and historical context (Toró et al., 2024; Trifunović, 2022). European studies have confirmed that age, education, and income play significant roles in vaccination decisions, supporting our observations (Cadeddu, 2023; Lupaşcu Moisi et al., 2022). However, the magnitude of some effects in our data—particularly the urban–rural gap and the income gradient—appears larger than what has been reported in Western European samples, suggesting that structural inequalities in healthcare access and information infrastructure amplify the influence of these variables in the Romanian context.

A key distinguishing factor is Romania’s chronic institutional distrust, fueled by historical experiences with health policies and exacerbated by poor crisis communication during the COVID-19 pandemic (Mărcău et al., 2022a; Vulpe & Vasile, 2023). In countries with well-developed healthcare infrastructures and efficient public communication, scientific information is typically disseminated quickly through verified channels, reducing the influence of fake news (Manolescu et al., 2022; D. Sandu, 2022). In Romania, cultural and socioeconomic barriers limit access to quality information, particularly in rural areas, where the population remains more vulnerable to conspiracy narratives (Citu et al., 2022). Differences in digital literacy levels further widen this gap, resulting in a form of vaccine hesitancy shaped not only by misleading messages but also by deeply rooted institutional skepticism (Akhavavn & Rask, 2022).

These cultural specificities have practical implications for intervention design. While international best practices in counter-misinformation communication can serve as a starting point, their implementation in Romania must account for the additional layer of institutional distrust. Collaboration between authorities, nongovernmental organizations, and trusted community figures is essential to overcome cultural barriers and increase vaccine acceptance (Evans et al., 2023; Luke et al., 2024; Macchiarelli & Giacon, 2021). Strengthening digital infrastructure and improving access to quality healthcare in rural areas are equally important structural prerequisites for reducing informational gaps and lowering vulnerability to fake news (Ban et al., 2024; Lister & Joudrey, 2022; Misra et al., 2024; Reichelt et al., 2023).

### 4.5. Limitations and Directions for Future Research

Several methodological limitations should be considered when interpreting these findings.

First, the online administration of the questionnaire introduces a systematic selection bias. Respondents were necessarily individuals with internet access and sufficient digital skills to complete an online survey (Dutwin & Buskirk, 2023; Gaia et al., 2025). This likely underrepresents older adults, rural populations, and individuals with lower education and income—precisely the demographic groups that our analyses identified as most susceptible to misinformation and most hesitant toward vaccination. Consequently, the actual prevalence of vaccine hesitancy and misinformation susceptibility in the general Romanian population may be higher than our estimates suggest. Furthermore, individuals who voluntarily participate in online surveys about vaccination may hold stronger pre-existing opinions (either pro- or anti-vaccination), potentially amplifying the observed associations between misinformation belief and vaccine hesitancy through self-selection effects (Eysenbach, 2004; Petrovčič et al., 2024).

Second, social desirability bias may have affected responses (Helbing & Krumpal, 2025; Kale et al., 2024). Given the polarized public discourse surrounding COVID-19 vaccination in Romania, respondents may have underreported their belief in fake news statements or overreported their vaccination acceptance to conform to perceived social norms. This would lead to conservative estimates of the true associations, meaning the actual influence of misinformation on vaccine hesitancy could be stronger than observed (Archambault et al., 2023; Wolter et al., 2022).

Third, the cross-sectional design precludes causal inference (Folotiya & Ngoma, 2024). While the findings reveal strong associations between misinformation exposure and vaccine hesitancy, the direction of this relationship cannot be definitively established (Barbieri et al., 2024; S. K. Lee et al., 2022). It is equally plausible that individuals who are already hesitant about vaccines selectively seek out and endorse misinformation that confirms their pre-existing attitudes (confirmation bias), rather than misinformation directly causing hesitancy (Piksa et al., 2024). The moderate effect sizes observed are consistent with misinformation being one of several contributing factors rather than a singular determinant (Maier et al., 2023; Pérez-Guerrero et al., 2024).

Fourth, the vaccination willingness measures for hypothetical pandemic scenarios (Q8–Q10) capture stated intentions rather than actual behavior (Conner & Norman, 2022; Fishman et al., 2024). Research on the intention–behavior gap consistently shows that self-reported willingness to vaccinate tends to overestimate actual uptake, particularly when the threat is abstract rather than immediate. Since respondents were asked to imagine pandemics of varying severity without experiencing real consequences, their responses may reflect idealized rather than realistic decision-making. This limitation is especially relevant for the severe pandemic scenario (Q10), where high reported willingness (e.g., 77.5% among the highest income group) may not translate into equivalent vaccination rates in practice. Consequently, the observed increase in willingness from Q8 to Q10 should be interpreted as reflecting the direction and relative magnitude of the severity effect rather than as precise predictions of future behavior (Atanasova et al., 2025; Gandhi et al., 2025).

Fifth, the binary coding of several demographic variables (education: pre-university vs. university; residence: rural vs. urban; gender: male vs. female) may mask meaningful within-group variation (Miao et al., 2024). For instance, the “pre-university” category encompasses individuals with only basic education as well as high school graduates, two groups that may differ substantially in their information-processing abilities and misinformation susceptibility (Bergen et al., 2023). Future studies should consider more granular categorizations (Royston et al., 2006).

Sixth, self-reported measures of belief in fake news may be affected by response biases beyond social desirability, including acquiescence bias (a tendency to agree with statements regardless of content) and order effects (Hill & Roberts, 2023). The FN1–FN15 items were all framed as conspiratorial claims, and the absence of reverse-coded items may have inflated the internal consistency of the misinformation scale without adequately capturing the nuances of individual belief (Elek et al., 2025).

Seventh, although the FN1–FN15 scale demonstrated excellent internal consistency, the instrument has not been formally validated against established misinformation or conspiracy belief scales. The absence of convergent validity evidence (i.e., correlation with validated measures such as the Vaccine Conspiracy Beliefs Scale; Shapiro et al., 2016, or the Misinformation Susceptibility Test; Maertens et al., 2024) and discriminant validity evidence (i.e., demonstrating that the scale measures misinformation susceptibility specifically rather than a broader construct such as general institutional distrust or conspiratorial ideation) means that some caution is warranted when interpreting the findings. It is possible that the associations observed between FN scores and vaccination attitudes partly reflect underlying distrust in institutions rather than misinformation endorsement per se. Future studies should assess the psychometric properties of this instrument more rigorously, including confirmatory factor analysis and validation against external criteria.

To address these limitations, future studies should adopt longitudinal designs to capture the dynamics of vaccination attitudes as epidemiological contexts evolve. A combination of quantitative and qualitative methods could provide a broader understanding of the psychosocial mechanisms underlying vaccine hesitancy. Expanding the sample through probability-based sampling methods and conducting cross-country comparisons would help identify Romania’s unique contextual factors and improve generalizability. Experimental and intervention-based research—particularly studies testing inoculation-based counter-messaging strategies against the specific fake news items identified in this study (FN4, FN11, FN14, FN15)—would be essential for validating the causal pathways suggested by our correlational findings and for guiding evidence-based public health policies (Maier et al., 2023; Pérez-Guerrero et al., 2024).

## 5. Conclusions

The results of this study confirm both hypotheses. Hypothesis H1—that a higher level of misinformation is associated with reluctance toward vaccination—is supported by consistent Spearman correlations indicating that higher fake news belief scores are associated with lower vaccine acceptance across all categories (mandatory, optional, and pandemic-related). Random forest models identified FN4 (“Vaccines cause autism”), FN11 (“Childhood vaccination weakens the immune system”), FN14 (“Natural immunity is always safer”), and FN15 (“Vaccinated children are prone to autoimmune diseases”) as the most influential predictors of vaccine hesitancy, while Spearman correlations confirmed these associations across all analyzed scenarios (all *p* < 0.001). Hypothesis H2—that sociodemographic factors are significantly associated with susceptibility to misinformation and vaccination decisions—is supported by both bivariate (ANOVA, chi-square) and multivariate analyses (logistic regression, random forest). Age, gender, place of residence, education level, and income all emerged as significant predictors of vaccination behavior, with gender consistently showing the largest effect sizes (Cramér’s V = 0.295 for mandatory vaccines; β = 1.433 for vaccine refusal in the multinomial model).

The study’s findings indicate that vaccine hesitancy in Romania is strongly associated with exposure to misinformation, particularly statements that challenge vaccine safety and promote the superiority of natural immunity. Our analyses indicated that greater belief in fake news is associated with significantly lower vaccine acceptance. Moreover, sociodemographic factors play a crucial role; younger individuals, those with higher education, and urban residents are more likely to accept vaccination, whereas rural populations and individuals with lower educational levels are more vulnerable to misinformation. These findings underscore the importance of a tailored communication strategy that considers the specific characteristics of different population segments.

Secondly, risk perception was found to be directly correlated with vaccination intent, with acceptance increasing significantly as pandemic scenarios became more severe. This finding suggests that when perceived risk is high, even individuals exposed to false information may be motivated to get vaccinated. Thus, this study highlights the need for clear and coherent public information campaigns that not only combat misinformation but also emphasize the concrete benefits of vaccination in the face of real threats.

These conclusions provide an empirical basis for the development of evidence-informed public policies aimed at increasing vaccine acceptance and improving the healthcare system’s response to future epidemiological crises. Taken together, the results underscore that misinformation does not merely affect individual vaccination decisions but also erodes the broader sense of social security—understood as public trust in the institutional capacity to protect against collective health threats—making integrated counter-misinformation strategies a priority for both public health and societal resilience.

## Figures and Tables

**Table 1:** Sociodemographic data of the participants.

Variables		18–25 Years	26–35 Years	36–45 Years	46–55 Years	56–65 Years
		%				
Gender	Male	7.9	11.6	45.5	28	6.8
	Female	28.2	20.9	34.3	13.1	3.2
Environment	Rural	38.2	18.6	27.2	14.3	1.4
	Urban	11.8	15.2	43.8	22.9	6.1
Level of education	Middle and high school	35	14.4	22.7	19.4	8.3
	University	13.4	16.2	44.2	21.5	4.4
Status	Private employee	8.5	18.6	46.1	22	4.6
	Budget employe e	40.1	8.9	25.4	19	6.4
Monthly Income	1000–3500 lei	57.2	16.7	11.4	9.6	4.8
	3501–5000 lei	14.6	17.4	38.2	23.6	6.1
	5001–7500 lei	5.9	14.5	48.1	26.4	4.8
	Over 7500 lei	1.6	15.4	53.7	24.1	5

**Table 2:** Participants’ trust in “Fake News” statements.

Statements	Rating 3 (Neutral) Yes %	Rating 4–5 (High Trust) Yes %
FN1—The Spanish flu was caused by experimental vaccines.	15.4	6.0
FN2—Ebola was intentionally spread to reduce Africa’s population.	14.4	9.2
FN3—AIDS was created in a laboratory for population control.	14.8	8.0
FN4—Mandatory vaccines included in the national immunization program cause autism.	12.0	8.3
FN5—A new pandemic would be a stage of the “Global Plan” to establish a world dictatorship.	14.3	10.4
FN6—Vaccines developed for a new pandemic will include nanobots to monitor vaccinated individuals.	13.3	6.5
FN7—Pharmaceutical companies will invent a new pandemic to sell medication at high prices.	15.5	14.7
FN8—The virus that will cause a new pandemic will be spread through airplane chemtrails to infect people.	13.9	7.4
FN9—Pharmaceutical companies and governments are hiding the fact that vaccines cause infertility.	14.9	12.0
FN10—Natural medicine and a healthy diet are sufficient to prevent and treat various diseases, making vaccines unnecessary.	16.0	13.3
FN11—Childhood vaccination destroys the immune system and makes children vulnerable to any other disease.	12.7	11.8
FN12—Repeated vaccination leads to the complete destruction of the immune system.	12.2	15.3
FN13—Vaccines are full of heavy metals and toxic substances that cause neurological diseases.	12.4	13.4
FN14—Natural immunity is always safer and more effective than any vaccine.	13.2	22.9
FN15—Vaccinated children are more prone to allergies and autoimmune diseases than unvaccinated children.	13.6	14.5

**Table 3:** Vaccination attitudes by key sociodemographic variables (simplified).

Variable	Category	Q2—Mandatory Vaccines (Yes %)	Q4—Optional Vaccines (Yes %)	Q6—COVID-19 Vaccine (Yes %)
Gender	Male	95.7	89.1	86.7
	Female	75.1	65.6	60.9
	Effect	χ^2^ = 87.67, *p* < 0.001, V = 0.295	χ^2^ = 79.5, *p* < 0.001, V = 0.281	χ^2^ = 86.93, *p* < 0.001, V = 0.294
Age	18–25	77.5	67.8	41.3
	46–55	93.4	86.8	87.3
	Effect	χ^2^ = 24.85, *p* < 0.001, V = 0.157	χ^2^ = 25.14, *p* < 0.001, V = 0.158	χ^2^ = 134.28, *p* < 0.001, V = 0.366
Environment	Rural	79.9	68.8	54.0
	Urban	87.9	80.7	80.2
	Effect	χ^2^ = 8.36, *p* = 0.004, V = 0.091	χ^2^ = 13.06, *p* < 0.001, V = 0.114	χ^2^ = 58.98, *p* < 0.001, V = 0.242
Income	1000–3500 lei	76.2	66.9	46.6
	Over 7500 lei	93.9	87.4	87.9
	Effect	χ^2^ = 42.54, *p* < 0.001, V = 0.206	χ^2^ = 44.71, *p* < 0.001, V = 0.211	χ^2^ = 138.15, *p* < 0.001, V = 0.371

Note. Q2 = trust in mandatory childhood vaccines; Q4 = trust in optional vaccines; Q6 = received the COVID-19 vaccine. Chi-square tests used all categories of each variable. Full data, including all age groups, education levels, and employment status, are reported in Supplementary Table S1.

**Table 4:** (**a**) Clustering and group differences in vaccine acceptance by fake news beliefs. (**b**) Predictive importance and correlations of fake news items with vaccine acceptance.

(**a**)				
**Analysis**	**Purpose**	**Mandatory Vaccines (Q2)**	**Optional Vaccines (Q4)**	
PCA + K-Means Clustering	Identifying groups based on FN beliefs and vaccination decisions	3 clusters: (1) High trust (M = 1.03), (2) Moderate skepticism (M = 1.24), (3) High skepticism (M = 1.66)	3 clusters: (1) High trust (M = 1.08), (2) Moderate skepticism (M = 1.32), (3) High skepticism (M = 1.71)	
ANOVA	Testing differences between clusters on sociodemographic variables	Age: F = 39.73, *p* < 0.001, η^2^ = 0.073; Gender: F = 84.18, *p* < 0.001, η^2^ = 0.144; Income: F = 24.62, *p* < 0.001, η^2^ = 0.047	Age: F = 42.61, *p* < 0.001, η^2^ = 0.078; Gender: F = 79.35, *p* < 0.001, η^2^ = 0.137; Income: F = 21.50, *p* < 0.001, η^2^ = 0.041	
Tukey HSD	Pairwise differences between clusters	Cluster 3 differs from Clusters 1 and 2 in age, income, and education (*p* < 0.001)	Cluster 3 differs from Clusters 1 and 2 in age, income, and gender (*p* < 0.001)	
(**b**)				
**Fake News Item**	**RF Importance (Q2)**	**RF Importance (Q4)**	**Spearman ρ (Q2)**	**Spearman ρ (Q4)**
FN4—Vaccines cause autism	0.0959	0.1023	ρ = 0.508, *p* < 0.001	ρ = 0.544, *p* < 0.001
FN11—Vaccination destroys immune system	0.0925	0.0976	ρ = 0.517, *p* < 0.001	ρ = 0.618, *p* < 0.001
FN15—Vaccinated children prone to allergies	0.0706	0.0754	ρ = 0.469, *p* < 0.001	ρ = 0.586, *p* < 0.001

Note. Cluster means represent average vaccination attitude scores (1 = full trust, 2 = no trust). η^2^ = SS_between/SS_total. Note: Only the three most influential fake news items are shown. Random Forest importance represents the mean decrease in accuracy. Full results for all FN1–FN15 items are available in Supplementary Table S2.

**Table 5:** Vaccination acceptance in the event of a new pandemic.

Variable	Category	Q8—Milder (Yes %)	Q9—Similar to COVID-19 (Yes %)	Q10—More Severe (Yes %)
Age	18–25	16.9	20.1	31.6
	26–35	31.2	46.8	51.8
	36–45	47.2	63.5	69.2
	46–55	55.3	67.1	74.1
	56+	46.1	59.6	65.3
Gender	Male	51.4	69.9	76.0
	Female	28.7	35.2	42.9
Environment	Rural	28.2	33.9	39.2
	Urban	44.3	59.1	66.4
Education	Middle/high school	28.8	38.8	44.4
	University	43.6	57.2	64.3
Status	Private employee	45.7	61.2	67.7
	Budget employee	28.6	34.7	42.6
Income	1000–3500 lei	18.5	21.5	30.8
	3501–5000 lei	40.4	48.8	53.9
	5001–7500 lei	47.5	62.1	66.4
	Over 7500 lei	50.6	70.1	77.5

Note. Q8 = willingness to vaccinate if a milder pandemic than COVID-19 emerged; Q9 = if a pandemic similar to COVID-19 emerged; Q10 = if a more severe pandemic emerged. Percentages represent respondents who answered “to a great extent” or “to a very great extent” (Ratings 4–5).

**Table 6:** Most influential fake news items on vaccination intentions across pandemic scenarios (simplified).

Analysis	Q8—Milder Pandemic	Q9—Similar to COVID-19	Q10—More Severe
K-Means Clustering	2 clusters: (1) Skeptical group (high FN scores), (2) Pro-vaccination group (low FN scores). Pattern consistent across all scenarios.	—	—
Kruskal–Wallis	H = 289.84, *p* < 0.001, ε^2^ = 0.288	H = 345.58, *p* < 0.001, ε^2^ = 0.343	H = 385.54, *p* < 0.001, ε^2^ = 0.383
RF—Top 3	FN14 (15.3%), FN13 (14.9%), FN12 (14.0%)	FN12 (14.7%), FN14 (11.7%), FN15 (11.7%)	FN14 (13.6%), FN11 (11.7%), FN15 (10.7%)
Spearman—Top 3	FN14 (ρ = −0.592), FN12 (ρ = −0.588), FN13 (ρ = −0.578)	FN12 (ρ = −0.638), FN13 (ρ = −0.62), FN14 (ρ = −0.617)	FN12 (ρ = −0.656), FN11 (ρ = −0.648), FN13 (ρ = −0.643)

Note. Random Forest importance values represent % contribution to predictive accuracy. All Spearman correlations were significant at *p* < 0.001. ε^2^ = (H − 1)/(N − 1) for the Kruskal–Wallis test. Full results for all FN1–FN15 items are reported in Supplementary Table S3.

**Table 7:** Multinomial logistic regression: predictors of vaccine type preference (reference = mRNA).

Predictor	Viral Vector (β [95% CI])	Inactivated/Subunit (β [95% CI])	No Vaccine (β [95% CI])
Age	0.091 [−0.112, 0.294]	−0.288 [−0.558, −0.017]	−0.188 [−0.347, −0.028]
Gender	0.254 [−0.176, 0.683]	0.599 [0.014, 1.183]	1.433 [1.087, 1.780]
Residence	−0.143 [−0.711, 0.426]	−0.518 [−1.172, 0.135]	−0.608 [−1.018, −0.197]
Education	0.099 [−0.507, 0.704]	−0.665 [−1.320, −0.010]	−0.503 [−0.937, −0.069]
Income	−0.095 [−0.289, 0.100]	−0.318 [−0.582, −0.053]	−0.389 [−0.543, −0.235]

Note. Model fit: McFadden R^2^ = 0.114, Nagelkerke R^2^ = 0.255, LR χ^2^ = 261.3, *p* < 0.001. N = 1005. Reference category = mRNA vaccine. Gender coded 1 = male; 2 = female. Residence coded 1 = rural; 2 = urban. Education coded 1 = pre-university; 2 = university. Top Random Forest variables by vaccine category: mRNA—FN11 (10.5%), FN15 (10.0%), FN14 (10.0%); viral vector—FN5 (7.2%), FN4 (4.7%), FN2 (4.0%); inactivated/subunit—FN15 (10.0%), FN12 (9.1%), FN5 (7.2%); no vaccine—FN14 (10.0%), FN12 (9.1%), FN5 (7.2%).

## Data Availability

https://figshare.com/articles/dataset/Future_pandemics_vaccination/27246765?file=53042507 (accessed on 1 April 2026).

## Supplementary Materials

The following supporting information can be downloaded at https://www.mdpi.com/article/10.3390/bs16040595/s1: Table S1: Full cross-tabulation of vaccination attitudes by sociodemographic variables; Table S2: Full analysis of the influence of fake news on vaccine acceptance (Q2 and Q4); Table S3: Full analysis of the effect of fake news on vaccination acceptance across pandemic scenarios (Q8–Q10).

## Appendix A. Questionnaire

SECTION 1. SOCIO-DEMOGRAPHIC DATA

Age range you belong to:

1. 18–25

2. 26–35

3. 36–45

4. 46–55

5. 56–65

Gender:

1. Male

2. Female

Your permanent residence is in:

1. Rural area

2. Urban area

Level of education:

1. Pre-university studies

2. University studies

Where do you work?

1. Private sector

2. Public sector

What is your monthly income?

1. 1000–3500 lei

2. 3501–5000 lei

3. 5001–7500 lei

4. Over 7500 lei

Q1. Which values guide your behavior?

1. Scientifically proven achievements over time

2. Religious ideas and concepts

Q2. Do you trust the mandatory vaccines administered during childhood (Hepatitis B vaccine, hexavalent vaccine, tuberculosis vaccine, etc.)?

1. Yes

2. No

Q3. Were you vaccinated during childhood with the mandatory vaccines?

1. Yes

2. No

Q4. Do you trust optional vaccines (flu vaccines, hepatitis vaccines, HPV vaccines, etc.)?

1. Yes

2. No

Q5. Have you been vaccinated with optional vaccines?

1. Yes

2. No

Q6. Have you received the COVID-19 vaccine?

1. Yes

2. No

Q7. If you were vaccinated against COVID-19, which type of vaccine did you choose?

1. mRNA-based vaccine (e.g., Pfizer-BioNTech, Moderna)

2. Viral vector vaccine (e.g., AstraZeneca, Johnson & Johnson, etc.)

SECTION 2. HYPOTHETICAL PANDEMIC SCENARIOS

Please rate from 1 to 5.

1—To a very small extent

2—To a small extent

3—Neutral

4—To a large extent

5—To a very large extent

Q8. If a new pandemic, milder than COVID-19, were to emerge, would you be willing to get vaccinated?

1	2	3	4	5

Q9. If a new pandemic, similar to COVID-19, were to emerge, would you be willing to get vaccinated?

1	2	3	4	5

Q10. If a new pandemic, more severe than COVID-19, were to emerge, would you be willing to get vaccinated?

1	2	3	4	5

Q11. Which type of vaccine would you choose if you opted for vaccination?

1. mRNA-based vaccine

2. Protein-based vaccine

3. Viral vector vaccine

4. I do not want to get vaccinated

SECTION 3. STATEMENT ASSESSMENT

Please rate the following statements from 1 to 5.

1—To a very small extent

2—To a small extent

3—Neutral

4—To a large extent

5—To a very large extent

FN1—The Spanish flu was caused by experimental vaccines.	1	2	3	4	5
FN2—Ebola was intentionally spread to reduce Africa’s population.	1	2	3	4	5
FN3—AIDS was created in a laboratory for population control.	1	2	3	4	5
FN4—Mandatory vaccines included in the national immunization program cause autism.	1	2	3	4	5
FN5—A new pandemic would be a stage of the “Global Plan” to establish a world dictatorship.	1	2	3	4	5
FN6—Vaccines developed for a new pandemic will include nanobots to monitor vaccinated individuals.	1	2	3	4	5
FN7—Pharmaceutical companies will invent a new pandemic to sell medication at high prices.	1	2	3	4	5
FN8—The virus that will cause a new pandemic will be spread through airplane chemtrails to infect people.	1	2	3	4	5
FN9—Pharmaceutical companies and governments are hiding the fact that vaccines cause infertility.	1	2	3	4	5
FN10—Natural medicine and a healthy diet are sufficient to prevent and treat various diseases, making vaccines unnecessary.	1	2	3	4	5
FN11—Childhood vaccination destroys the immune system and makes children vulnerable to any other disease.	1	2	3	4	5
FN12—Repeated vaccination leads to the complete destruction of the immune system.	1	2	3	4	5
FN13—Vaccines are full of heavy metals and toxic substances that cause neurological diseases.	1	2	3	4	5
FN14—Natural immunity is always safer and more effective than any vaccine.	1	2	3	4	5
FN15—Vaccinated children are more prone to allergies and autoimmune diseases than unvaccinated children.	1	2	3	4	5
