# Supplementary material for: Psychosocial and Social Security Risks Linked to Vaccine Misinformation in Romania: Implications for Vaccination Acceptance and Public Policy
Source: Behav Sci (Basel). 2026 Apr 16;16(4):595. doi: 10.3390/bs16040595 (PMC13113947; doi:10.3390/bs16040595)
Supplement: Supplementary file 1 [file behavsci-16-00595-s001.zip › behavsci-4187482-supplementary.pdf]

Supplementary Materials

Supplementary Table S1. Full cross-tabulation of vaccination attitudes by sociodemographic variables

Variable	Category	Q2 (Yes %)	Q3 (Yes %)	Q4 (Yes %)	Q5 (Yes %)	Q6 (Yes %)
**Age**	18–25	77.5	94.2	67.8	41.9	41.3
	26–35	81.2	96.8	72.5	60.6	73.7
	36–45	87.9	98.2	80.7	59.6	82.5
	46–55	93.4	99.5	86.8	69.9	87.3
	56+	88.4	100	76.9	51.9	78.8
**Gender**	Male	95.7	98.7	89.1	66.2	86.7
	Female	75.1	96.5	65.6	49.4	60.9
**Environment**	Rural	79.9	94.7	68.8	49.7	54.0
	Urban	87.9	98.4	80.7	60.8	80.2
**Education**	Middle/high school	80.5	95.0	71.1	51.6	58.3
	University	87.5	98.3	79.8	60.0	78.4
**Status**	Private employee	88.4	97.9	81.2	63.2	79.8
	Budget employee	80.6	97.1	70.6	46.2	61.6
**Income**	1000–3500 lei	76.2	95.1	66.9	43.1	46.6
	3501–5000 lei	82.5	97.7	70.2	53.3	73.0
	5001–7500 lei	84.8	98.9	79.4	58.9	81.6
	Over 7500 lei	93.9	98.5	87.4	68.9	87.9

Note. Q2 = Do you trust the mandatory childhood vaccines? Q3 = Were you vaccinated as a child with mandatory vaccines? Q4 = Do you trust optional vaccines? Q5 = Have you been vaccinated with optional vaccines? Q6 = Have you received the COVID-19 vaccine?

Supplementary Table S2. Full analysis of the influence of fake news on vaccine acceptance (Q2 and Q4)

Analysis	Purpose	Results for Q2 (Mandatory Vaccines)	Results for Q4 (Optional Vaccines)
PCA + K-Means	Identifying groups based on FN beliefs and vaccination decisions	3 clusters: (1) High trust (Q2 = 1.03), (2) Moderate skepticism (Q2 = 1.24), (3) High skepticism (Q2 = 1.66)	3 clusters: (1) High trust (Q4 = 1.08), (2) Moderate skepticism (Q4 = 1.32), (3) High skepticism (Q4 = 1.71)
ANOVA	Checking differences between clusters	Age ($F = 39.73$, $p < 0.001$, $\eta^2 = 0.073$), Gender ($F = 84.18$, $p < 0.001$, $\eta^2 = 0.144$), Income ($F = 24.62$, $p < 0.001$, $\eta^2 = 0.047$)	Age ($F = 42.61$, $p < 0.001$, $\eta^2 = 0.078$), Gender ($F = 79.35$, $p < 0.001$, $\eta^2 = 0.137$), Income ($F = 21.50$, $p < 0.001$, $\eta^2 = 0.041$)
Tukey HSD	Pairwise differences	Cluster 2 differs from Clusters 0 and 1 in age, income, and education ($p < 0.001$)	Cluster 2 differs from Clusters 0 and 1 in age, income, and gender ($p < 0.001$)
Random Forest	Most influential variables	FN4 (0.0959), FN11 (0.0925), FN15 (0.0706)	FN4 (0.1023), FN11 (0.0976), FN15 (0.0754)
Spearman Correlation	FN–vaccine trust relationship	FN4 ($\varrho = 0.508$), FN11 ($\varrho = 0.517$), FN15 ($\varrho = 0.469$), all $p < 0.001$	FN4 ($\varrho = 0.544$), FN11 ($\varrho = 0.618$), FN15 ($\varrho = 0.586$), all $p < 0.001$

Supplementary Table S3. Full analysis of the effect of fake news on vaccination acceptance across pandemic scenarios (Q8–Q10)

Analysis	Purpose	Q8	Q9	Q10
K-Means	Group identification	2 clusters: Skeptical (high FN) vs. Pro-vaccination (low FN)	—	—
Kruskal–Wallis	Group differences in vaccination intent	$H = 289.84$, $p < 0.001$, $\varepsilon^2 = 0.288$	$H = 345.58$, $p < 0.001$, $\varepsilon^2 = 0.343$	$H = 385.54$, $p < 0.001$, $\varepsilon^2 = 0.383$
ANOVA	Demographic differences	Age: $F = 20.97$, $\eta^2 = 0.077$; Gender: $F = 96.21$, $\eta^2 = 0.088$; Residence: $F = 28.37$, $\eta^2 = 0.028$; Education: $F = 17.04$, $\eta^2 = 0.017$; Income: $F = 29.29$, $\eta^2 = 0.081$	Age: $F = 27.56$, $\eta^2 = 0.099$; Gender: $F = 142.43$, $\eta^2 = 0.124$; Residence: $F = 47.94$, $\eta^2 = 0.046$; Education: $F = 25.52$, $\eta^2 = 0.025$; Income: $F = 49.56$, $\eta^2 = 0.129$	Age: $F = 24.79$, $\eta^2 = 0.09$; Gender: $F = 151.91$, $\eta^2 = 0.132$; Residence: $F = 52.83$, $\eta^2 = 0.05$; Education: $F = 29.29$, $\eta^2 = 0.028$; Income: $F = 54.61$, $\eta^2 = 0.141$
Random Forest	Most influential variables	FN14 (15.3%), FN13 (14.9%), FN12 (14.0%), FN5 (9.9%), FN15 (8.6%)	FN12 (14.7%), FN14 (11.7%), FN15 (11.7%), FN11 (9.0%), FN13 (8.9%)	FN14 (13.6%), FN11 (11.7%), FN15 (10.7%), FN13 (9.5%), FN12 (8.9%)
Spearman	FN–vaccination intent correlations	All FN1–FN15 significant ($p < 0.001$). Top: FN14 ($\varrho = -0.592$), FN12 ($\varrho = -0.588$), FN13 ($\varrho = -0.578$)	All significant ($p < 0.001$). Top: FN12 ($\varrho = -0.638$), FN13 ($\varrho = -0.62$), FN14 ($\varrho = -0.617$)	All significant ($p < 0.001$). Top: FN12 ($\varrho = -0.656$), FN11 ($\varrho = -0.648$), FN13 ($\varrho = -0.643$)
